# Antiviral monoclonal antibody cocktails as a modern weapon in combating pandemics

**DOI:** 10.4155/tde-2021-0079

**Published:** 2021-11-02

**Authors:** Dingjiang Liu, Mohammed Shameem

**Affiliations:** ^1^Formulations Development & Delivery Technology, Preclinical Development, Regeneron Pharmaceuticals, Inc., 777 Saw Mill River Road, Tarrytown, NY 10591–6707, USA

**Keywords:** antibody cocktail, antiviral, coformulation, formulation, high concentration, monoclonal antibody, pandemic, REGEN-COV, SARS-CoV-2

Following the emergence of the SARS-CoV-2 global pandemic in early 2020, over 20 biotechnology and pharmaceutical companies around the world initiated clinical testing of over 30 novel monoclonal antibody (mAb) molecules [1], subsequent to identification of the viral spike protein target and characterization of its genetic sequence. While many of these companies moved their single best mAb candidate into clinical development, a few companies – such as Regeneron – focused exclusively on the development of antibody cocktails, a proven strategy for mitigating the emergence of viral escape mutants, which is particularly problematic with rapidly mutating viruses such as SARS-CoV-2.

AbCellera/Eli Lilly and Company was the first to bring a single antibody, LY-COV555 (bamlanivimab), into clinical testing on 28 May 2020. On 9 November, bamlanivimab was granted an emergency use authorization (EUA) by the US FDA for the treatment of mild to moderate coronavirus disease 2019 (COVID-19) in adults and pediatric patients [2]. However, as anticipated, variants of the initial strain of SARS-CoV-2 emerged and in October 2020, the Beta variant (B.1.351, also called South Africa variant) emerged and was found to be resistant to bamlanivimab [3]. By March 2021, with the prevalence of viral variants increasing around the world, the US government halted distribution of bamlanivimab due to loss of efficacy [4].

On 21 November 2020, Regeneron’s REGEN-COV two-mAb cocktail consisting of casirivimab and imdevimab was issued an EUA for the treatment of COVID-19 in adults and pediatric patients [5]. Although some of the viral variants were resistant to casirivimab, the two-antibody cocktail maintained its ability to neutralize the virus and to date has remained efficacious against all known SARS-CoV-2 variants of concern [3]. The superior efficacy of REGEN-COV has led to a significant increase in its use in the US, with full approval being granted in many other countries, including Japan and the UK. The success of REGEN-COV (Ronapreve^®^ brand name outside USA), as well as the previous approval of a 3-antibody cocktail INMAZEB^®^ for treatment of Ebola, confirmed the superiority of the cocktail approach for combating rapidly evolving pandemics like COVID-19.

## Coformulation development of mAb cocktails

Compared with the development of single-antibody formulations, development of antibody cocktail formulations presents a number of additional challenges. The first and most obvious challenge is the impact on manufacturing due to the need to supply the multiple mAbs that comprise the cocktail. To this end, it is essential to have a clear operational strategy in hand at the outset of development in order to meet anticipated, early demand stemming from EUA; one such strategy could involve formation of global collaborations to substantially augment production capacity.

With regard to formulation and drug product development, having a high concentration liquid formulation is critical, not only to reduce the overall product volume but to enable both intravenous (iv.) and subcutaneous (sc.) administration, a critical but often underappreciated option. Incorporating options for both sc. and intramuscular (im.) administration into early formulation design and clinical studies will substantially reduce overall development time while improving access to the drug following approval [6]. It must be remembered that iv. infusion administration is time consuming and cumbersome, and typically requires special facilities that reduce potential for disease transmission during administration, thus limiting patient capacity at these facilities and slowing the process of treating patients. Given that single doses of mAb drugs used for treating infectious diseases typically exceed 500 mg [7–9], high concentration liquid formulations (>100 mg/ml) are essential for enabling sc. or im. drug products.

High-concentration coformulation approaches offer clear advantages for the end user, especially as it pertains to dose preparation and administration. However, there are many unique considerations and challenges involved in the development of high-concentration solutions of coformulated drug products. In order to determine interactions that may result when antibodies are coformulated, biophysical characterization of the individual antibodies, both alone and in the coformulated solution, must be performed and include measurements of viscosity, protein interactions (kD, B22) and thermal/conformational stability by differential scanning calorimetry. Information obtained from these studies is invaluable in guiding critical decisions during formulation development, particularly with respect to the stability of the coformulation.

Another important consideration lies in quickly developing proper analytical methods for drug product release and stability testing. For coformulations, a method to qualify the amount and ratio of the mAbs that provides sufficient separation to monitor the attributes of each antibody is required [10,11]. Developing these methods quickly is technically challenging given that the selected mAbs are often similar in size, charge and hydrophobicity. For example, conventional methods, such as size-exclusion ultra-performance liquid chromatography alone are often incapable of providing quantitative information for the homo- and hetero-aggregates and require extended analysis using advanced mass spectrometric methods.

Coformulation also requires an additional manufacturing process for mixing the individual antibodies. The coformulated drug substance can either be produced immediately prior to filling the drug product at the fill-finish site or combined at a drug substance manufacturing facility prior to filling. Ideally, both process options should be defined during development in order to provide the flexibility needed to maximize manufacturing capacity.

While the challenges in developing mAb cocktail formulation may seem overwhelming, they are not insurmountable to experienced formulators, as the rapid development of INMAZEB and REGEN-COV by Regeneron has proven.

One unexpected challenge in developing antibody cocktails for managing the emergence of drug-resistant viral variants of SARS-CoV-2 was the need to modify the existing formulation by replacing individual antibodies that have lost efficacy with newly identified antibodies. This challenged initial thinking regarding the primary advantage of coformulations. This experience suggests that in the early phases of pandemics, co-administration of the individual antibody formulations may initially be preferable to coformulated drug products as the former allows the opportunity to select combinations of antibodies with the widest spectrum of activity against rapidly emerging viral escape mutants prior to engaging in the additional effort of developing the coformulated drug product.

## Conclusion

mAb, particularly cocktails of multiple antibodies, are playing a significant role in managing the current COVID-19 pandemic. Millions of doses of mAbs, including Regeneron’s REGEN-COV and other antibodies developed by Lilly, Vir/GSK and AZ, have significantly decreased the risk of emergency visit or hospitalization and saved many lives [12]. As a result of these admirable efforts, tremendous experience has been gained in multiple aspects of pharmaceutical product development, including lead molecule selection and design, formulation development, manufacturing and supply and drug preparation and administration, as well as governmental management and social acceptance. In the future, it is anticipated that the experienced so gained will allow for development of even more effective mAb drugs and in particular, antibody cocktails, leading to more rapid containment of infectious diseases and resultant, improved patient outcomes.
